# What’s NORMal for Fracking? Estimating Total Radioactivity of Produced Fluids

**DOI:** 10.1289/ehp.123-A186

**Published:** 2015-07-01

**Authors:** Lindsey Konkel

**Affiliations:** Lindsey Konkel is a New Jersey–based journalist who reports on science, health, and the environment.

Naturally occurring radioactive materials (NORM) found in liquid wastes from hydraulic fracturing are an emerging environmental health concern.^1^ The heavily drilled Marcellus Shale, for example, contains isotopes of radium, polonium, and lead.^2^^,^^3^ However, the few studies that have focused on NORM in fracking wastewater (produced fluids) have reported on a single element—radium.^4^^,^^5^ In this issue of *EHP*, researchers estimate total reactivity for a mixture of isotopes present in liquid fracking waste from the Marcellus Shale.^3^

Decay chains—the series of transformations that radionuclides undergo until a stable isotope is produced—are important in planning for the treatment, management, and disposal of radioactive waste.^6^ Long-lived radioactive elements (such as ^226^radium) transform into shorter-lived decay products (such as ^210^lead and ^210^polonium). Some decay products approach the same radioactivity concentration as the parent radionuclide in a matter of days, while others do so over many decades.

**Figure:**
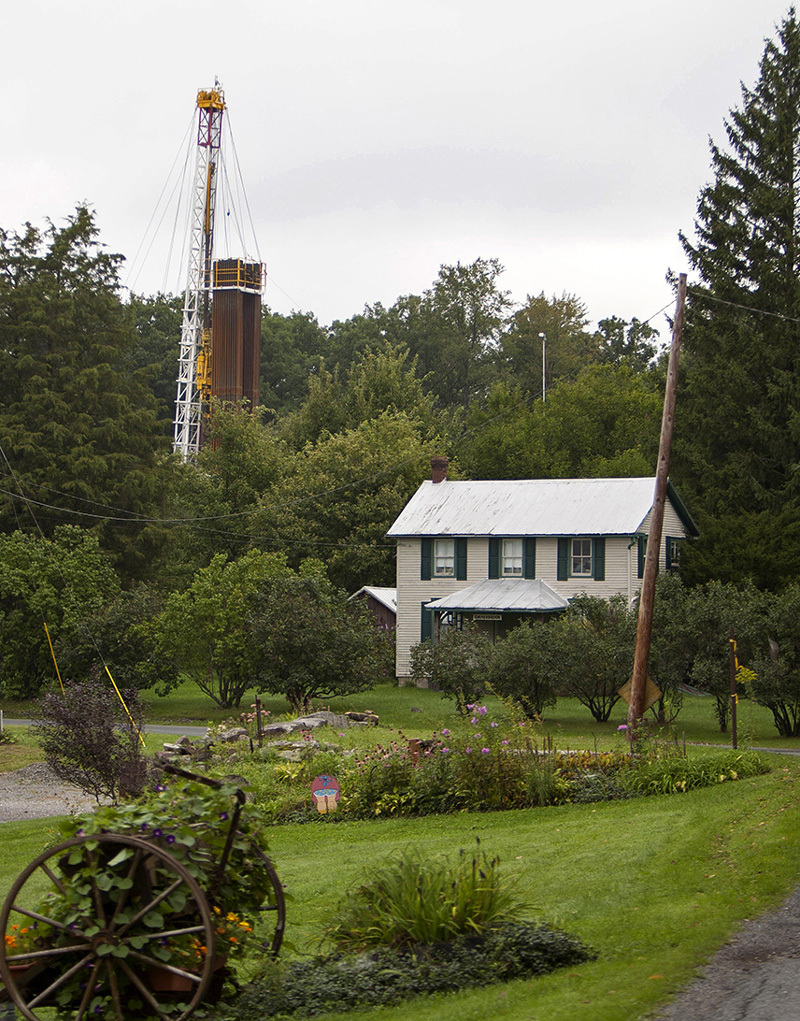
In heavily drilled areas like the Marcellus Shale region, fracking activities often take place near homes and workplaces. © Robert Nickelsberg/Getty Images

For the study, researchers at the University of Iowa modeled how concentrations of radionuclides in fracking waste, and thus levels of radioactivity, would change over time. The researchers obtained a sample of fracking wastewater collected from the Marcellus Shale region in 2012 and sealed in a 200-liter drum. They used gamma and alpha spectrometry to measure existing levels of radionuclides in the sealed sample. Using models to account for radioactive decay and ingrowth (increasing concentrations of decay products), the researchers estimated how the total radioactivity concentration of the produced fluids would change in the foreseeable future.

“Much of the work we present here was used to develop and validate a rapid radioactivity measurement method for a federal agency,” says lead author Andrew Nelson, a Presidential Graduate Research Fellow and PhD candidate in human toxicology. “We had to have a controlled environment so we could be sure what we were measuring and confident in our analyses. If we left the cap open always, the values would be changing unpredictably.”

Under the closed-system conditions, the researchers estimated that the radioactivity concentration would increase by a factor of more than five within 15 days as a result of radioactive ingrowth. They measured an increase in the decay products ^210^lead and ^228^thorium in the closed system, and they predicted that radioactivity would continue to increase for more than 100 years with the formation of the decay products ^210^lead and ^210^polonium.

“We know so little about the extent to which these contaminants represent major health concerns,” says James Burch, an associate professor in the University of South Carolina Department of Epidemiology and Biostatistics. “This study lays the foundation for a more detailed investigation of the health impacts of radioactive waste that may be generated from fracking.” Burch was not involved in the current study.

Because fracking activities and wastewater disposal often take place in close proximity to where people live and work, there’s a potential for human exposure, according to Burch. “The technology is vastly outpacing what we know about the health effects,” he says.

A few cases of radioactive contamination outside of wastewater treatment facilities have been documented. In one notable example, researchers studied wastewater effluent discharged by the Josephine Brine Treatment Facility in Pennsylvania. In stream sediments near the facility, the researchers measured concentrations of ^226^radium that were approximately 200 times higher than would normally be expected.^7^

The authors of the current study conclude that future studies and risk assessments should include radium decay products in assessing the potential for environmental contamination from fracking”^5^ Nelson calls the current research a “conversation starter” that aims to inform risk assessment and waste handling. “If you don’t have an accurate measurement of the radiochemical parameters, you can’t have an accurate dose assessment,” he says.

While the experimental design represents a starting point, it’s unclear how applicable the projections would be under real-world conditions where fluids are being moved around, handled, and exposed to the air, says Arthur Rose, an environmental geochemist and professor emeritus at Pennsylvania State University, who was not involved in the research. “In this study you have a worst-case scenario in which all the decay products remain in the water,” Rose says. “A more likely scenario would be that some of the radon would escape into the air. Future studies need to look at radon release.”

In ongoing field studies, Nelson and colleagues are applying new methods they developed to real-world scenarios. One study is looking at how radioactivity concentrations of naturally occurring radionuclides and their decay products change over time at a wastewater treatment facility that handles unconventional drilling waste.
